# Impact of Adopting a Multidisciplinary Care Delivery Model on Treatment Effectiveness and Outcomes in Stage III Melanoma Patients at a Community-Based Academic Institution: A Retrospective Study

**DOI:** 10.7759/cureus.79703

**Published:** 2025-02-26

**Authors:** Zeeshan Hafeez, Aidan Johnson, Nicole Nester, Hariharasudan Mani, Aaron Blackham, Alyson McIntosh, Lauren Davis, Angela Miller, Alexandra Bauman, Morgan Horton, Suresh Nair

**Affiliations:** 1 Hematology and Medical Oncology, Lehigh Valley Topper Cancer Institute, Allentown, USA; 2 Graduate Medical Education, Geisinger Commonwealth School of Medicine, Scranton, USA; 3 Graduate Medical Education, Philadelphia College of Osteopathic Medicine, Philadelphia, USA; 4 Surgical Oncology, Lehigh Valley Topper Cancer Institute, Allentown, USA; 5 Radiation Oncology, Lehigh Valley Topper Cancer Institute, Allentown, USA

**Keywords:** disease-free survival (dfs), health care delivery, multidisciplinary care (mdc), nurse navigation, overall survival (os)

## Abstract

Purpose

Stage III melanoma represents a high-risk patient population, highlighting the need to implement strategies to prevent the onset of metastatic disease. This study evaluates the impact of systems of care, incorporating multidisciplinary care (MDC), nurse navigation, clinical trials, and early anti-PD1 immunotherapy use, on outcomes for this patient population.

Patients and methods

We conducted a retrospective chart review to evaluate the impact of an updated melanoma MDC program implemented at our institution in late 2016. This program facilitated access to nurse navigation, along with early PD-1 immunotherapy use and clinical trial enrollment, with the expectation of improved outcomes for our melanoma patients. Data were collected from 121 stage III melanoma patients treated at our institution between 2014 and 2019, divided into two cohorts: 2014-2016 (n = 66) and 2017-2019 (n = 55). Primary outcomes included three-year disease-free survival (DFS) and overall survival (OS). Secondary outcomes included disease recurrence rates and melanoma-related mortality.

Results

Implementation of an MDC model led to a significant increase in immunotherapy use, multidisciplinary visits, and nurse navigation. These combined factors resulted in a 30% increase in three-year DFS (from 48% to 78%, p = 0.001) and a 17% increase in three-year OS (from 63% to 80%, p = 0.04). There was a 30% decrease in stage III melanoma mortality (39% vs. 9%, p < 0.001).

Conclusion

Systematic sub-specialized care, nurse navigation, access to clinical trials, MDC care, and rapid adoption of advancements in the standard of care led to improved patient outcomes at our institution.

## Introduction

Cutaneous melanoma, derived from the malignant transformation of melanocytes, is the most aggressive form of skin cancer [1]. It is the fifth most prevalent cancer in the United States and represents a significant health concern [2]. Geographic variations exist, with Australia/New Zealand exhibiting the highest incidence rates, followed by Western Europe and North America [3]. Over the past few decades, there has been a steady rise in melanoma incidence, with an annual increase of 3%-7% in the White population [4], particularly among older patients [5]. This trend is attributed to factors such as increased sun exposure and changing environmental conditions [6].

Stage III melanoma represents a high-risk stage characterized by the spread of malignant cells beyond the primary site to regional lymph nodes [7-9]. Surgical excision remains the cornerstone of initial treatment [8,9]. Adjuvant radiation therapy (RT) has been used to reduce recurrence risk, but its effectiveness is debated due to limited overall survival (OS) benefits [10]. PD-1 and CTLA-4 are key immune checkpoint receptors that suppress T-cell activity, allowing melanoma cells to evade immune detection. Blocking these receptors enables the immune system to better detect melanoma cells. The emergence of immunotherapy, including PD-1 inhibitors (e.g., nivolumab and pembrolizumab) and anti-CTLA-4 agents (e.g., ipilimumab), has marked a paradigm shift in the management of stage III melanoma [1,11]. Ipilimumab was the first agent granted FDA approval in the adjuvant setting in 2015, based on its superiority over a placebo in recurrence-free survival for resected stage III melanoma [12]. Nivolumab received FDA approval in December 2017, based on longer recurrence-free survival compared to ipilimumab in the Checkmate 238 trial [13]. Pembrolizumab was approved in February 2019, based on improved disease-free survival (DFS) compared to the placebo in the Keynote 054 trial [14]. The current standard of care for stage III melanoma includes single-agent PD-1 inhibitor therapy for one year following surgical management, or a combination of BRAF/MEK inhibitors for BRAF-mutated patients [1]. Despite these advancements, the risk of disease recurrence remains a significant concern.

In 2016, Lehigh Valley Health Network (LVHN) joined the Memorial Sloan Kettering Cancer Center Alliance, aligning with best practices in cancer care, expanding access to clinical trials, and fostering collaboration with leading researchers. This partnership facilitated the adoption of standardized, evidence-based treatment protocols and care pathways, significantly enhancing the quality, consistency, and delivery of cancer care.

In 2014, LVHN introduced multidisciplinary care (MDC) models for breast, thoracic, and prostate cancers, followed by the inclusion of melanoma in 2016. The MDC team comprises sub-specialists, including melanoma-dedicated medical oncologists, surgical oncologists, and radiation oncologists, as well as nurse navigators, clinical trial coordinators, social service providers, and genetic counselors - all collaborating to develop individualized treatment plans. MDC meetings were held at least twice a month to ensure coordinated and comprehensive care.

Nurse navigators played a vital role in coordinating care, providing support, and serving as a centralized point of contact for patients and their families. To evaluate the benefits of this model, we conducted a single-center retrospective review of stage III melanoma patients, assessing the impact of MDC, nurse navigation, and other interventions on outcomes. This study aims to identify approaches beyond standard treatments to improve care delivery and survival in stage III melanoma patients.

## Materials and methods

We conducted a comprehensive retrospective observational chart review at LVHN to assess the impact of care delivery systems on clinical outcomes in patients diagnosed with stage III melanoma. We included adult patients aged 18 years or older with pathologically and radiographically confirmed stage III melanoma who were eligible for melanoma-directed treatments, including surgical resection, adjuvant RT, or immunotherapy. Inclusion criteria required complete medical records, including imaging, follow-up data, and pathology reports, for all patients treated at our institution between 2014 and 2019. Patients with concurrent malignancies or end-stage non-cancer diagnoses were excluded to minimize confounding factors influencing the immune system, treatment responses, and overall patient outcomes.

After assessing eligibility, we reviewed the medical records of 121 stage III melanoma patients over a six-year period (2014-2019). Patients were stratified into two cohorts based on treatment timing: Cohort 1 (2014-2016, n = 66) and Cohort 2 (2017-2019, n = 55). Key data points included adjuvant immunotherapy use, engagement with the melanoma MDC model, and involvement with nurse navigation services. We also collected data on surgical resection of the primary melanoma lesion and adjuvant RT. Patient demographic and clinical characteristics, including age, gender, primary treatment type, and enrollment in clinical trials, are summarized in Table 1.

**Table 1:** Patient characteristics

Characteristics	2014-2016 (N = 66)	2017-2019 (N = 55)
Sex		
Males	32 (49%)	33 (60%)
Females	34 (51%)	22 (40%)
Age groups		
20-29	2 (3%)	3 (5%)
30-39	5 (8%)	2 (4%)
40-49	8 (12%)	4 (7%)
50-59	10 (15%)	12 (22%)
60-69	17 (26%)	14 (25%)
70-79	11 (17%)	9 (16%)
>80	13 (19%)	11 (20%)
Received surgery	65 (98%)	55 (100%)
Received radiation	14 (21%)	11 (20%)
Received adjuvant immunotherapy	28 (43%)	33 (60%)
Received adjuvant interferon	17 (26%)	0
Clinical trial participation	11 (17%)	1 (2%)
Multidisciplinary care (MDC) participation	35 (53%)	40 (74%)
Nurse navigation	36 (55%)	46 (83%)

Three-year DFS and OS were chosen as primary outcomes to assess treatment efficacy and long-term prognosis, as most recurrences in stage III melanoma occur within this timeframe. To provide further insight into the effectiveness of our care delivery model, we calculated secondary outcomes, including disease recurrence rates and the percentage of deaths attributable to melanoma.

A comparative analysis was conducted across the two time periods to evaluate trends and variations in key data points. Percentage changes were calculated for each data point between the two periods. To assess intervention effects, three-year DFS (time from primary treatment initiation to disease recurrence) and three-year OS (time from primary treatment initiation to death from any cause) were calculated for both cohorts. We applied Chi-square tests to determine the statistical significance of differences between three-year OS and three-year DFS. Standard statistical measures, including relative risk (RR), odds ratio (OR), absolute risk reduction (ARR), and number needed to treat (NNT), were calculated to quantify the outcome analysis between the two groups. A comparative analysis of care delivery component utilization before and after 2016 was conducted to identify trends and their effects on patient outcomes. We considered cause-specific mortality to ensure accurate survival outcome assessment. Due to the retrospective design of the study, formal adjustments for confounding variables, such as age and comorbidities, were not conducted in this analysis.

## Results

**Figure 1:**
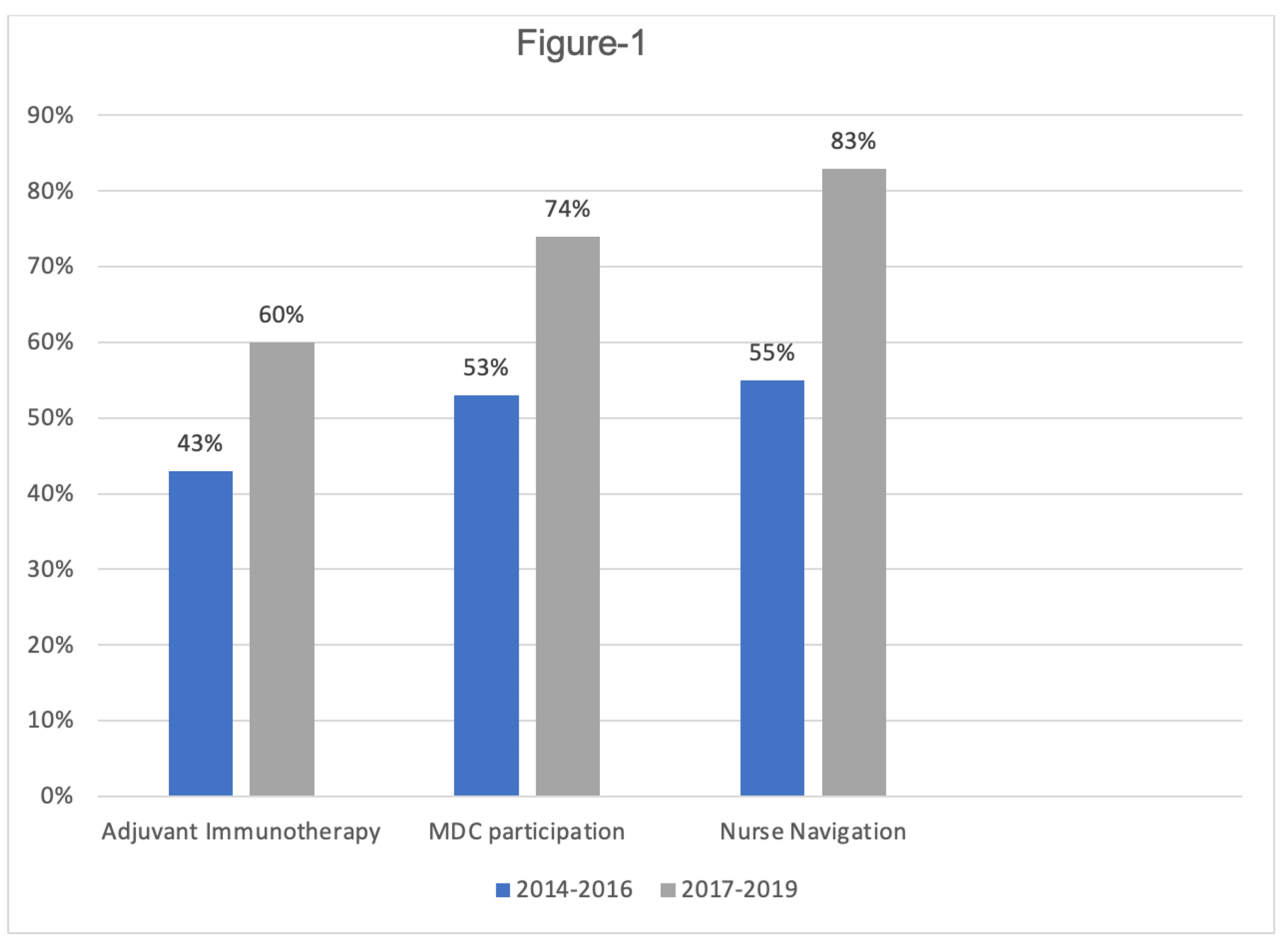
Trends in key interventions across two cohorts: adjuvant immunotherapy use, MDC participation, and nurse navigation MDC, Multidisciplinary care

Key data elements, including demographic and clinical characteristics of the two cohorts, are summarized in Table 1. At the data cut-off points between cohorts, adjuvant immunotherapy utilization increased by 17%, from 28 patients (43%) in Cohort 1 to 33 patients (60%) in Cohort 2. Additionally, five patients (17%) in Cohort 1 were participating in checkpoint inhibitor trials, which led to their approval (Figure 1). Nearly all patients in both cohorts underwent primary treatment with surgical resection. The utilization of adjuvant radiation was quite comparable between the two cohorts, with 14 patients (21%) in Cohort 1 and 11 patients (20%) in Cohort 2 (Table 1).

There was a 21% increase, from 35 patients (53%) to 40 patients (74%), in MDC appointments between cohorts, while nurse navigation involvement increased by 28%, from 36 patients (55%) to 46 patients (83%) (Figure 1).

The three-year DFS rate increased from 48% to 78% (p = 0.001; RR: 1.61, 95% CI: 1.22-2.12; OR: 3.81, 95% CI: 1.83-7.92), representing a 30% improvement. The ARR of 29.7%, indicating a nearly 30% reduction in the risk of disease recurrence at three years, with an NNT of 4, meaning that for every four patients treated, one additional patient was prevented from experiencing disease recurrence. Similarly, the three-year OS rate significantly improved, increasing from 63% to 80% (p = 0.04; RR: 1.29, 95% CI: 1.02-1.63; OR: 2.44, 95% CI: 1.05-5.65), reflecting a 17% increase. The ARR of 17.9%, indicating an 18% reduction in the risk of death at three years, with an NNT of 6, meaning that for every six patients treated, one additional death was prevented (Table 2).

**Table 2:** Principal statistical results DFS, Disease-free survival; OS, Overall survival

Outcome	2014-2016 (n = 66)	2017-2019 (n = 55)	Relative risk (95% CI)	Odds ratio (95% CI)	p-value
3-yr DFS	48% (32)	78% (43)	1.61 (1.22-2.12)	3.81 (1.83-7.92)	0.001
3-yr OS	63% (41)	80% (44)	1.29 (1.02-1.63)	2.44 (1.05-5.65)	0.04

**Figure 2:**
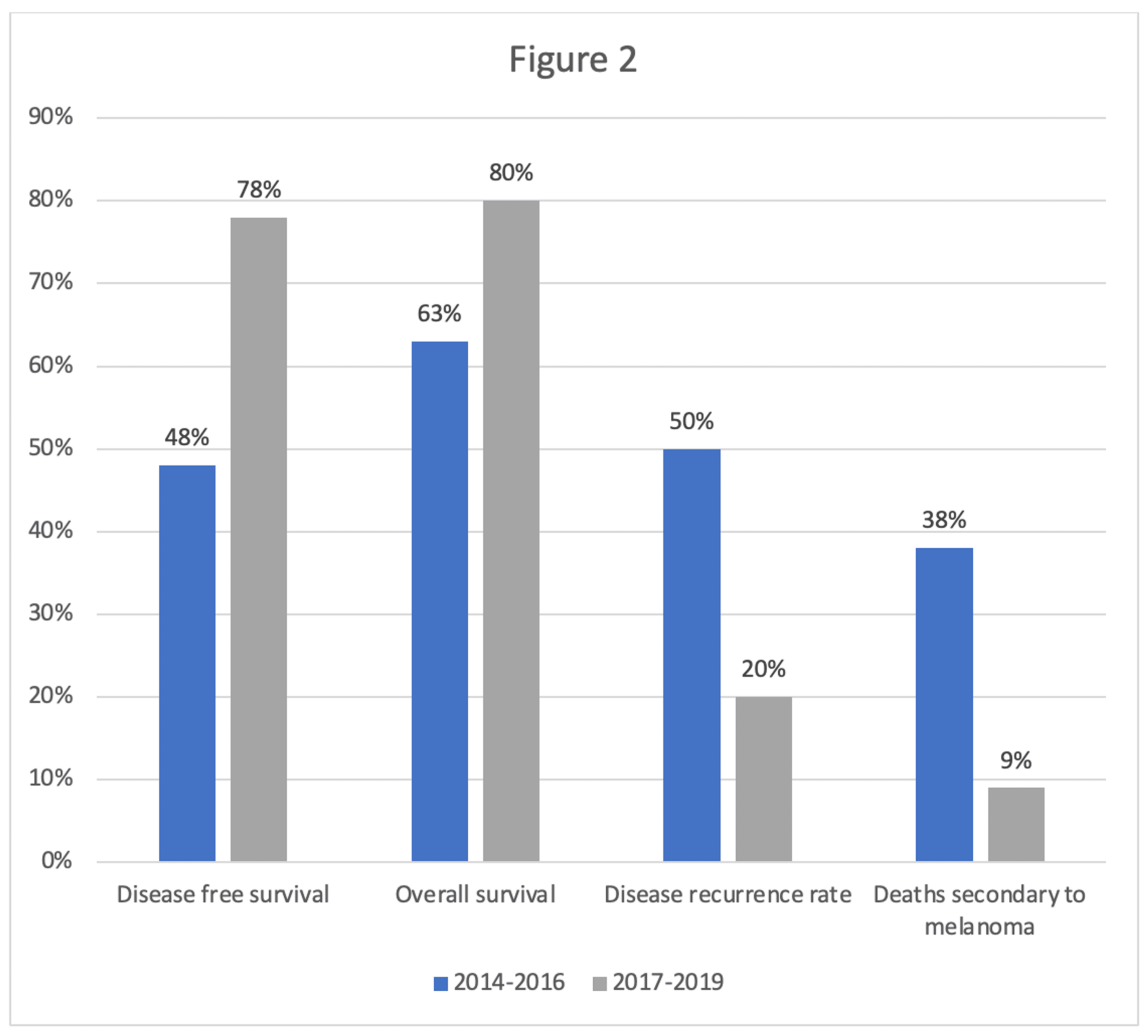
Primary and secondary outcome results

These improvements coincided with increased immunotherapy use (from 43% to 60%), MDC visits (from 53% to 74%), and nurse navigation involvement (from 55% to 83%), as well as a substantial increase in checkpoint inhibitor therapy through clinical trials (from 17% to 60%). Secondary outcomes analysis revealed a 30% decrease in stage III melanoma mortality rates, with the percentage of deaths attributed to melanoma decreasing from 39% to 9% (p < 0.001) (Figure 2).

The trends observed in key interventions across the two study cohorts are summarized in Figure 1, while Figure 2 summarizes the results for the primary and secondary outcomes.

## Discussion

Despite significant advancements in the detection and treatment of stage III melanoma, it remains a major health challenge, underscoring the need for improved multidisciplinary coordination and the timely integration of novel therapies to enhance patient outcomes. Effective management requires integrated risk models that incorporate a range of clinical and molecular factors [1]. Multidisciplinary cancer care, which promotes collaboration among healthcare providers, has been recognized as a best practice since 1995 [15,3]. The use of an MDC model, combined with nurse navigation services, can significantly enhance healthcare delivery and improve patient outcomes. MDC is a patient-centered approach that unites healthcare teams to address the needs of disadvantaged patients, leading to better outcomes across various cancer types [16].

Nurse navigators play a vital role in addressing the unique needs of cancer patients and their families by adopting a personalized approach, recognizing that "no one size fits all," and providing guidance, advocacy, and emotional support throughout the cancer diagnosis and treatment journey [17]. They schedule appointments, discuss treatment options, and connect patients with support services such as counseling. By addressing barriers (e.g., transportation or financial challenges), ensuring understanding of treatment plans, and coordinating MDC, nurse navigators improve therapy adherence, reduce treatment delays, and enhance overall outcomes.

However, challenges persist in implementing this model, including limited data on its impact and the need to integrate research and innovation to further enhance patient outcomes [18]. Therefore, additional research is necessary to quantify the benefits and positive impacts of such interdisciplinary models on patient care.

The upward trends observed in our study, including increased MDC appointments, utilization of nurse navigation services, and use of adjuvant immunotherapy, reflect early shifts in our institution’s approach to melanoma care delivery. The 30% increase in three-year DFS rates is particularly noteworthy, demonstrating the efficacy of the implemented care models and treatment strategies. Additionally, the 30% decrease in melanoma-related mortality highlights the real-world impact of therapeutic advancements in stage III melanoma, driven by the early adoption of anti-PD1 therapy from clinical trials to standard of care. Notably, adjuvant nivolumab and pembrolizumab were FDA-approved during the second period based on pivotal trial results.

A limitation of our study is the retrospective nature of the analysis and the inability to distinguish the specific contributions of adjuvant immunotherapy, MDC care, and nurse navigation. Future prospective randomized controlled trials (RCTs) could address this by isolating the individual and combined effects of these interventions. For example, patients could be randomized into different treatment arms, such as standard care without MDC and nurse navigation versus standard care with MDC and nurse navigation participation.

We believe the observed improvements in DFS, OS, and mortality strongly suggest a positive impact from the implemented interventions. The consistency of these findings across multiple endpoints further supports the validity of our conclusions. Moreover, OS in our patients was better than the national averages, as per the National Cancer Institute database at that time, for regional disease of less than 70% [19], highlighting the important contribution of MDC care for the early adaptation of modernized treatments and nurse navigation to increase compliance, leading to OS benefits and reduced mortality in stage III melanoma patients. While our findings are specific to LVHN, they align with broader evidence supporting the effectiveness of integrated care models.

## Conclusions

A specialized care approach, incorporating nurse navigation services, clinical trial access, and MDC, was associated with significant improvements in patient outcomes in our melanoma program. Our findings suggest that integrated care models can enhance melanoma care and reduce mortality. The success of interdisciplinary programs at LVHN in improving patient satisfaction and healthcare delivery highlights the potential for their adoption in community hospitals, academic medical centers, and cancer care centers to optimize outcomes for melanoma and other high-risk cancers.
